# Disagreements in physical activity monitor validation study guidelines create challenges in conducting validity studies

**DOI:** 10.3389/fdgth.2022.1063324

**Published:** 2023-01-10

**Authors:** Myles W. O’Brien, Liam P. Pellerine, Madeline E. Shivgulam, Derek S. Kimmerly

**Affiliations:** ^1^ School of Physiotherapy (Faculty of Health) & Division of Geriatric Medicine (Faculty of Medicine), Dalhousie University, Halifax, NS, Canada; ^2^ Geriatric Medicine Research, Dalhousie University & Nova Scotia Health, Halifax, NS, Canada; ^3^ Division of Kinesiology, School of Health and Human Performance, Faculty of Health, Dalhousie University, Halifax, NS, Canada

**Keywords:** harmonization, accuracy, wearable technology, frameworks, statistical analysis

## Introduction

In recent years, multiple groups consisting of researchers and/or industry partners have developed and published guidelines/frameworks designed to be a helpful resource for those planning an activity monitor validation study (1–8). However, problems arise for those planning validation studies when inconsistencies among these recommendation papers exist. This poses a challenge in the design and analysis stages for researchers, as well as for journal reviewers in evaluating whether strict adherence to published guidelines were followed.

The purpose of this opinion article was to highlight some of the consistent and divergent recommendations for conducting activity monitor validation studies. The following recent articles (i.e., within the last ∼4 years) form the basis of this document (1–8). This will not be an exhaustive list of (in)consistencies, but rather a focus on aspects such as statistical analysis and interpretation, which we feel are among the most salient to conducting a validity study.

## How to statistically assess the validity of activity monitors?

The interpretation of study outcomes relies heavily on the results of statistical tests implemented that compare the device of interest to a criterion measure. While simple correlations are typically implemented to determine if the values observed between the comparator-criterion are associated with each other, the determination of validity from correlations alone is insufficient (1). The interpretation of results, and thus conclusions drawn, may vary depending on the specific statistical tests implemented. This poses an issue when guidelines suggest divergent statistical tests be utilized. This point is particularly evident with current guidelines, as the Towards Intelligent Health and Well-Being Network of Physical Activity Assessment (INTERLIVE) group recommends Bland-Altman analyses (i.e., fixed/proportional biases) and mean absolute percent error (MAPE) (2, 3, 5), whereas the Consumer Technology Association (CTA) recommends MAPE only (6, 7), and Welk et al. (1) recommends additional tests (i.e., comparison of means, correlations, and equivalence tests). The Welk et al. (1) guidelines specifically emphasized the inclusion of equivalence testing, which was the only suggested statistical test recommended by Kozey-Keadle et al. (4) to assess validity.

Determining a device to be “not different” to a criterion (e.g., *via* ANOVA or fixed bias) does not necessarily imply that two measures are statistically equivalent. Equivalence testing (9–11) has been utilized to determine whether or not two measures provide statistically equivalent outcomes (12). A primer on equivalence testing is presented elsewhere (13). While challenges to implementing equivalence testing exist, such as the establishment of thresholds to denote “equivalence” (14, 15), the overarching idea of establishing whether two measures statistically produce values within an acceptable level of error seems pertinent to device based validation studies. Despite the heterogeneous recommendations, it is our position that equivalence testing be implemented alongside bias testing (i.e., Bland-Altman), difference of means, and MAPE. Conducting these detailed statistical analyses may help better characterize the validity of the measure of interest, permit between-study comparisons, and aid in the establishment of acceptable error levels with further use.

## How to interpret validation study results?

While we position that the same statistical battery should be conducted for research and commercial monitors, the acceptable level of error may be higher for commercial monitors, depending on the overarching objective. In agreement with Argent et al. (5), if improving health is the primary objective and users are depending on commercial devices for general proxies of step accumulation or energy expenditure, a less strict threshold of error is likely reasonable. However, specifics on what constitutes a monitor as valid/invalid are challenging to discern.

Bland-Altman analyses provide useful information regarding whether a comparator measure consistently over- or under-predicts activity as a function of the average of the comparator and criterion (i.e., fixed bias) or if the magnitude of the error changes as a function of the average (i.e., proportional bias) (16, 17). Limits of agreement (LoA) are calculated as 1.96 × standard deviation (SD) of the difference between the comparator-criterion and are encouraged by INTERLIVE to be the primary determinant of validity (2, 3, 5). Welk et al. (1) also highlighted the potential utility of Bland-Altman analyses. While we agree that Bland-Altman analyses are useful, there are also limitations of using this test as the primary determinant of whether monitors are valid or not. While LoA describe the range of error between measures, it is unclear how this value should be interpreted. Specifically, the magnitude of LoA that constitutes a small, medium, and large error have not been established. It is plausible that with the same LoA, two researchers may interpret the validity of the device differently. An “appropriate” LoA likely depends on the study protocol, criterion measure, minimal error of interest, and outcome metric utilized. Nevertheless, there is not a harmonized guide to assist researchers to best interpret their outcomes. Similarly, MAPE provides an indication of individual level error, but the interpretation of this outcome is unclear. CTA recommends heart rate monitors have a MAPE <10% to be deemed valid based on standards established for electrocardiography (7). Whether it is reasonable or not to extrapolate this to commercial wearable monitors is arguable. For monitors to be a valid measure of step counts, the CTA recommends <20% MAPE (6), whereas INTERLIVE recommends <5% for activity trackers to be used in clinical trials but <10%–15% for general public use (3). The justification for these thresholds is unclear. Whether a MAPE of <5%, <10%, <20%, or <50% is indicative of a low individual level error is unclear and may be interpreted differently depending on what researchers perceive as an acceptable level of error considering the context of their study (e.g., a larger error may be more acceptable in an uncontrolled free-living protocol vs. more controlled laboratory protocol). Therefore, this introduces human error and biases into the interpretation of findings. This is problematic for putting forth consistent conclusions across research labs, evaluating between-monitor validity, and the amalgamation of studies for meta-analyses.

Consistent with some guidelines (1, 4), we encourage that equivalence testing be conducted and that the equivalence zone required for the two measures to be deemed statistically equivalent reported. This avoids the use of arbitrary *a priori* thresholds (e.g., ±10% or ±20%) that produce dichotomous outcomes that are sensitive to minor deviations in threshold selection (14). Specifically, a review on the topic demonstrated that a 5% change in threshold selection altered the conclusions of 75% and 71% of validation studies in children/youth and adults, respectively (14). In the absence of clinically acceptable equivalence zones, we also recommend that researchers consider reporting the zone required for the measures to be deemed equivalent as a percentage and/or as a proportion of the SD (e.g., 0.5 SD). Examples for calculating exact equivalence zones as a relative percentage or as a proportion of SD can be found elsewhere (18–20).

For activity monitor validation studies, analyses will likely continue to be heterogeneously implemented without more guidance of how results should be interpreted. This is especially important when minor effects are statistically significant and/or multiple statistical tests produce divergent outcomes. Effect sizes should be consistently reported. From a guidelines point-of-view, it would be beneficial to provide some consistent insight into what are acceptable, evidence-based LoA/MAPE and how individual researchers should interpret the results of these statistical tests when making claims about device validity.

## How many people should be collected?

The number of participants recruited for a validation study should ideally be based on a power calculation. Sample sizes should consider the effect size of interest, study design, study hypothesis, planned statistical tests, and resources of the study (e.g., equipment available, personnel involved, etc.) (21). It should be appreciated that minor differences may be statistically significant with enough participants. Accordingly, sample size calculations that rely on difference-based hypothesis testing (e.g., between-monitor *t*-test, one sample *t*-test to a value of zero, ANOVAs, etc.) are estimating the number of participants needed for that difference to be statistically significant. Based on this logic, it should be unsurprising when statistically significant differences are observed when the number of participants recruited are based on a difference-based calculation. As outlined in (1), we agree that if the hypothesis is that a monitor will be equivalent to a criterion, then the sample size calculation should be based on equivalence testing [see (13, 22) for tips on how to do this].

While other guidelines do not provide a specific number of participants needed for validity studies (1, 4, 6, 7), the INTERLIVE group recommends a sample size calculation based on the comparator-criterion difference (5) or a sample of 45 people if insufficient evidence exists (2, 3, 5). If 45 people have multiple observations (e.g., repeated treadmill stages that get progressively faster), it is more probable that a minor fixed/proportional bias will be statistically significant. While we do not dismiss the use of Bland-Altman analyses, we feel that: (1) more emphasis should be placed on the magnitude of the fixed/proportional bias, (2) we need a better understanding of what are acceptable magnitude of biases, (3) we should not blindly follow statistically significant results, and (4) that the results of the Bland-Altman should be interpreted alongside other statistical tests. In addition, this provokes further thought on the use of 1 data point vs. multiple datapoints per participant. Guidelines that provide researchers with clear instructions of how to deal with multiple observations, evidence-based optimal sample size calculations, and analytical processing strategies would facilitate the adoption of a consistent process and help move the field in the same direction.

## What is consistent between recommendation guidelines?

**Figure 1:**
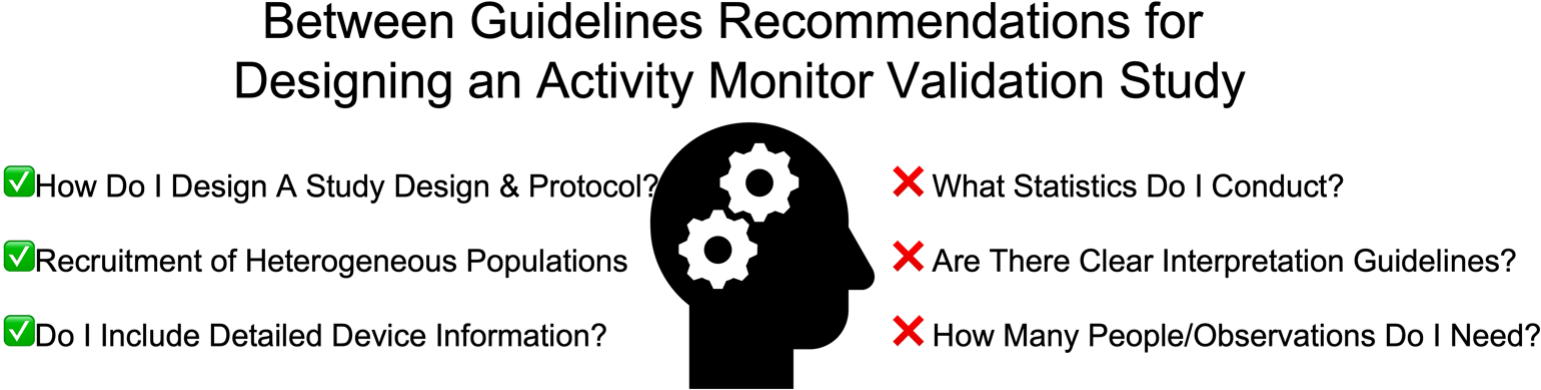
Aspects that are consistent and divergent between physical activity monitor validation recommendations (1–8). This figure depicts the considerations that may impact an individual researcher planning on conducting a monitor validation study, with checkmarks and “x” marks indicating consistent and divergent recommendations, respectively.

Figure 1 presents the inconsistent and consistent recommendations between guidelines for activity monitor validation studies that apply to researchers conducting these types of studies.

The INTERLIVE group (2, 3, 5), CTA (6–8), and Kozey-Keadle et al. (4) provide useful frameworks for step-by-step procedures in designing validation studies. The first phase of the framework presented by Kozey-Keadle et al. (4) outlines mechanical testing to determine the validity/reliability of the devices underlying electronics in the absence of the variability introduced by human movement (4). This highly controlled testing is essential for ensuring acceleration characteristics respond as expected to a known stimulus (e.g., *via* wheels, orbital shakers, etc.), and that the responses are the same when the identical stimulus is applied.

Groups (2–5, 8) advocate for the initial implementation of laboratory-based validation studies involving highly controlled protocols and accurate criterion measures (e.g., video-recorded steps). If validated in laboratory conditions, then the transition to semi-structured settings that involve general task instructions (e.g., household chores) are warranted, these tasks may include aspects of personal care, household chores, work/education, and leisure activities (4). The final stage of validating a device is to test it in a free-living or naturalistic setting, where there is the least amount of experimental control but is the setting where devices are typically used (8). CTA provides important considerations for validation studies in naturalistic settings (8). This spectrum from most-to-least researcher control and least-to-most external applicability provides a useful guide for the design of physical activity monitor validation testing protocols.

It is likely that the device of interest is being studied for use among a heterogeneous general population. Therefore, the recruitment of a diverse group of participants should also be encouraged, with considerations for age, sex, race, body mass index, occupational status, physical activity level, atypical gait patterns, etc. (2–6). The INTERLIVE groups idea of a checklist may serve as a useful resource in establishing minimum participant characteristic and analytical strategy reporting (e.g., epoch length, device version, sampling rate, etc.) (2, 3, 5).

## Conclusion

Inconsistencies across different recommendation guidelines in the same field of study create challenges. In the absence of guidelines that recommend the same thing, it is unclear which specific procedures researchers should adhere to. We highlight such challenges and pose further questions that may be of interest to help develop and/or revise future recommendations. The information presented in this opinion article is a call to action for wearable researchers to acknowledge these inconsistencies and work towards recommendations that advance the activity monitoring field. Rather than researchers establishing recommendations in silos with their colleagues, the establishment of a set of harmonized guidelines that incorporates a more extensive number of experts across the world (e.g., using the Delphi method) is needed to adopt a consistent set of experimental and analytical guidelines. Such procedures would establish a stronger consensus-based guidelines and may be a major step towards establishing evidence-based guidelines.
